# Current Hospital Topics

**Published:** 1906-11-03

**Authors:** 


### HOSPITAL ADMINISTRATION.

### CONSTRUCTION AND ECONOMICS.

### CURRENT HOSPITAL TOPICS.

#### The London Temperance Hospital.

We are glad to be able to record that the recommendation of King Edward's Hospital Fund to provide a more commodious and better equipped out-patient department at this hospital has borne fruit, and that the foundation-stone of a new out-patient hall was laid by Lady Strong on the 25th ultimo. It is proposed to carry out, first, that portion of the plan which includes a waiting hall to seat 200 out-patients, medical and surgical consulting and examining rooms, and a theatre for minor operations, with other necessary accommodation, at an estimated cost of about £11,000. The entire scheme will give, in addition, isolation accommodation, observation wards, a museum, and a lecture room for nurses, at a cost of about £25,000. We welcome this evidence of determination on the part of the management to complete and perfect the Temperance Hospital, because it is one of the most economically administered, and, from the patient's point of view, one of the most comfortable of metropolitan hospitals. Of the first £11,000 some £4,500 has still to be raised, so that all friends of temperance and economical management have here an opportunity to contribute wisely to a hospital which deserves every support and sympathy from the public.

#### A Scottish Expert.

Dr. Donald J. Mackintosh during his term as medical superintendent has brought the Western Infirmary, Glasgow, to a high pitch of efficiency. It is pleasant to be able to record that he is now recognised as the Scottish expert in matters of hospital administration and construction. We recently called attention to the report he made on the Perth Infirmary, and to the important changes and improvements, including probably the rebuilding of the whole institution, which may result from the recommendations he has made. The new out-patient department at the Western Infirmary has many novel features, and is so perfect in many respects that it is likely to form the unit which will probably be copied in most of its details in future modern hospitals. He is at present engaged upon the plans of a new maternity department, and has also been called in in connection with the plans for an addition to the Cancer Hospital and an extension of the MacAlpine Nursing Home. Not only has Dr. Mackintosh shown marked ability in matters of construction and administration, but as an administrator his system of control is so complete, as to ensure the maximum of economy. Our American cousins who travel all over the world are very quick to recognise ability, and we are glad to know that the fame of Dr. Mackintosh has spread to the United States. We have reason to think that his services may be sought in the administration of one of the large hospitals in that country, should they not be previously engaged by one of the greater metropolitan hospitals.

#### Up-to-date Methods.

In the colonies it is becoming increasingly the practice to issue a small monthly paper at the price of one penny, which contains a full account of the most interesting items in the work of the hospital whose cause it advocates, and so has a circulation not only among the patients and staff, but amongst the governors also. We believe that British hospitals would be well advised to consider this method of extending a knowledge of their work, and increasing their popularity. The patients alone at a great hospital are amply sufficient to ensure a circulation large enough to enable such a paper to be issued monthly, and to be self-supporting on its circulation alone. It is no part of the object of a publication of this kind to take advertisements, but it should be possible to issue a four-page paper full of interesting items, which could not fail to be popular, and to yield a definite revenue by increasing the number of annual subscribers and extending the area of givers to each institution, which has the ability and foresight successfully to carry out this most modern of methods for raising money. A relatively small but important cottage hospital at Springhill Mines, Nova Scotia, by the issue quarterly of a paper such as we have indicated, has succeeded in a very poor district in securing an endowment fund, the revenue from which is nearly equal to the total expenditure each year. Nothing more remarkable in recent hospital development has been brought to our notice than the success which has attended the self-denying efforts of the Rev. W. Chas. Wilson, to whom the success of this hospital is mainly due. He has shown literary ability, too, in the production of the quarterly paper published for the friends and devoted to the interests of the All Saints' Cottage Hospital.

#### Royal Naval Hospitals.

Captain Alfred Carpenter, R.N., has issued a letter from Sanderstead, Surrey, in which he states that our naval defenders are subjected "to shameful neglect when they are struck down by fever or accident and sent to hospital." His remarks do not, of course, apply to Haslar Hospital, which is one of the oldest of British hospitals, and is deservedly notable for the high efficiency of its administration and for the wonderful way in which the defects of so old a building have been remedied by modern improvements and structural changes.

Captain Carpenter really seems to have been aroused by his knowledge of one of the naval sick quarters situated on the south coast, where "there are no female trained nurses, and the male nurses are the ordinary half-trained young sick berth attendants," whom he describes as "mere hobbledehoys at the best." He instances the case of a patient suffering from typhoid fever, a disease the results of which depend largely upon the skill exhibited by the nurses in charge. This patient was ordered by the physician to be washed, and Captain Carpenter describes the method pursued by the sick berth attendant as follows:—"First he pulled all the bedclothes back to the patient's feet; then he turned the patient's pyjamas up to his knees; he then left the room, leaving the door and window open, in order to get the water. When remonstrated with he lost his temper and slammed the door so that the building shook." In another case one of the patients rang the bell at night for a whole hour without any one replying, although the bell could be heard plainly in the corridors. Captain Carpenter is justified in stating that it is a great defect and a mistaken economy not to provide the very best accommodation and means for speedily restoring to active service all naval officers and men who may unfortunately require hospital treatment. We venture to call the attention of the Medical-Director-General of the Navy to the statements of Captain Alfred Carpenter, so that an inspection may be made of the smaller naval sick quarters in various parts of the country, with a view to the speedy application of improved methods in the nursing and administration.

